# Impact of propofol sedation on the diagnostic accuracy of hepatic venous pressure gradient measurements in patients with cirrhosis

**DOI:** 10.1007/s12072-021-10261-z

**Published:** 2021-10-26

**Authors:** Fahim Ebrahimi, David Semela, Markus Heim

**Affiliations:** 1 Division of Gastroenterology and Hepatology, Clarunis University Center for Gastrointestinal and Liver Diseases, 4031 Basel, Switzerland; 2 Division of Gastroenterology and Hepatology, Kantonsspital, St. Gallen, 9007 St. Gallen, Switzerland

**Keywords:** HVPG, Sedation, Diagnostic accuracy, Cirrhosis, Portal hypertension

## Abstract

**Background:**

Measurement of the hepatic venous pressure gradient (HVPG) is the gold standard to evaluate the presence and severity of portal hypertension. The procedure is generally safe and well tolerated, but nevertheless, some patients demand for sedation. However, it is unknown whether propofol sedation would impair the accuracy of portal pressure measurements.

**Methods:**

This is a prospective observational cohort study including cirrhotic patients with suspected portal hypertension undergoing invasive measurement of HVPG. Measurements of HVPG were performed in awake condition as well as under sedation with propofol infusion.

**Results:**

In total, 37 patients were included. Mean HVPG in awake condition was 15.9 mmHg (IQR 13–19) and during sedation 14.1 mmHg (IQR 12–17). While measures of free hepatic vein pressure (FHVP) were not altered after propofol sedation (*p* = 0.34), wedged hepatic vein pressure values (WHVP) decreased in an average by  2.05 mmHg (95% CI − 2.46 to − 1.16; *p* < 0.001) which was proportional to the magnitude of HVPG. In 31 out of 37 patients (83.8%), portal hypertension with HVPG ≥ 12 mmHg was found. Under sedation with propofol, two patients (5.4%) with borderline values would have been incorrectly classified as < 12 mmHg. After adjustment for the average difference of − 10%, all patients were correctly classified. Intraclass correlation coefficient between HVPG measurement in awake condition and under propofol sedation was 0.927 (95% CI 0.594–0.975).

**Conclusions:**

Propofol sedation during HVPG measurements is generally safe, however it may lead to relevant alterations of HVPG readings.

**Supplementary Information:**

The online version contains supplementary material available at 10.1007/s12072-021-10261-z.

## Introduction

Liver cirrhosis is a continuum, ranging from a compensated to a decompensated stage, as defined by the occurrence of decompensating events such as variceal haemorrhage, ascites, or hepatic encephalopathy [1]. Since decompensation of cirrhosis is known to be associated with a markedly reduced life expectancy, it is pivotal to detect patients at risk.

Aside from systemic inflammation, mitochondrial dysfunction, oxidative stress and metabolic changes, portal hypertension has been described as one of the main determinants for the development of decompensated cirrhosis. The magnitude of portal hypertension can be ascertained via measurement of hepatic venous pressure gradient (HVPG) [2–4]. Complications of portal hypertension, i.e. development of esophageal varices, arise when HVPG increases above 10 mmHg, being termed as ‘‘clinically significant portal hypertension’’ (CSPH) (Baveno VI) [5]. Though, severe clinical events of decompensation in form of bleeding, ascites, or hepatic encephalopathy are known to develop when HVPG increases over a threshold value of 12 mmHg [6, 7]. Among patients with CSPH, those with a HVPG ≥ 16 mmHg are at increased risks of hepatic decompensation and mortality [8, 9].

Several non-invasive techniques have been proposed as surrogate markers, yet none of them has accomplished to replace the invasive HVPG measurement which still is the gold standard for evaluation of portal hypertension [5, 10]. Despite its minimally invasive nature and very low rates of adverse clinical events, many patients are anxious and unwilling to undergo the procedure and demand for sedation [11]. While propofol sedation is known to be generally safe in the ambulatory setting, it has relevant hemodynamic effects with vasodilatation, reduction of arterial blood pressure and cardio-depression [12]. However, it is not known whether propofol sedation would significantly alter measures of HVPG and lead to inaccurate conclusions in the guidance of the treatment of portal hypertension. Hence, the aim of this study was to investigate the hemodynamic effects of propofol on HVPG measures in cirrhotic patients with suspected portal hypertension.

## Materials and methods

### Patients

Adult patients (age > 18 years) with cirrhosis and suspected portal hypertension undergoing HVPG measurement between 01/2007 and 12/2009 were prospectively included in this study. Patients with inability to obtain a reliable HVPG measurement due to vein-to-vein collaterals, portal-vein thrombosis, or known allergy to propofol were excluded from the trial. All patients provided written informed consent. The study was approved by the Ethics Committee of the University Hospital of Basel (EKNZ) and performed in accordance with the ethical guidelines of the Declaration of Helsinki [13].

### Procedure

The HVPG measurements were performed by experienced hepatologists following a standard operating procedure [14]. In brief, after local anesthesia (mepivacaine 1% subcutaneously), an 8F catheter introducer (Fogarty 12TLW807F, Edwards Lifesciences, Irvine CA, USA) was inserted in the right jugular vein under ultrasound guidance (HA710, EZU-MT28-S1, Hitachi Medical Corporation, Tokyo, Japan) using the Seldinger technique. The guidance catheter was placed in a hepatic vein under fluoroscopic control, either the middle or right hepatic vein. Then, a balloon-tipped catheter was inserted under fluoroscopic guidance replacing the guidance catheter to acquire the free hepatic venous pressure (FHVP) with freely floating tip of the catheter and the wedged hepatic venous pressure (WHVP) after inflation of the balloon. The adequacy of occlusion was checked by injection of a small amount of contrast medium after balloon inflation under fluoroscopic control. All pressures were taken as triplicates after a stable value was obtained. HVPG values were calculated as the difference between the mean values of WHVP and FHVP measures.

After the first measurement in awake condition, all patients underwent a second measurement with sedation using intravenous application of propofol. Infusion rates of propofol were titrated to achieve and maintain a moderate level of sedation that allowed the patient to tolerate the procedure with minimal to mild pain while maintaining adequate cardiorespiratory function. The moderate level of sedation was defined as a Richmond Agitation-Sedation Scale score of − 3 points [15]. During sedation, all patients received nasal oxygen at flow rate of 2 L/min and were monitored using continuous pulse oximetry, electrocardiogram, and serial blood pressure measurements every 2–5 min. Sedation and monitoring of patients status during the sedation were carried out by specialized nurses experienced in application of intravenous sedation.

In some cases, after completing both measurements of the HVPG, transjugular liver biopsy was performed via the same catheter introducer sheath placed in the right internal jugular vein.

### Study outcomes

The primary aim was to assess the diagnostic accuracy of HVPG measurements with and without propofol sedation. Proportions of correctly or wrongly classified patients were compared between both assessment strategies. Secondary aims included assessment of the diagnostic accuracy after error adjustment.

### Statistical analysis

Unless stated otherwise, categorical variables are expressed as number (percentage) and continuous variables were reported as mean ± standard deviation (SD).

Estimates of the effect sizes and corresponding 95% confidence intervals (CI) were determined using linear regression. Receiver operating characteristic (ROC) analyses were performed to analyse the discriminative power to identify patients with CSPH (10 mmHg) or a threshold of ≥ 12 mmHg. CSPH prognosticates the formation of esophageal or gastric varices and is per se associated with a worse prognosis [2]. The cutoff at 12 mmHg defines an increased risk of variceal rupture [16]. Correlation analyses were performed calculating Spearman’s rho (*r*). Paired Student’s *t* test was applied for comparisons of normally distributed continuous data. A Bland–Altman Plot was created for agreement analysis. Measurement differences outside of the 95% CI limits of the Bland–Altman Plot were analyzed to identify potential predisposing factors for mismatch. All *p* values are two sided. All statistical analyses were performed using STATA, version 15.1 (StataCorp LLC).

## Results

### Patient characteristics

Demographic and baseline clinical characteristics of the 37 patients included in the study are presented in Table 1. The median age was 56 years, the median BMI 24.2 kg/m^2^, and the majority were male (62%). Main causes of cirrhosis included alcoholic liver disease (54%) and chronic hepatitis C infection (22%). Around half of the patients were classified as CHILD–Pugh class A or B, respectively, with only one patient classified as C. Patients had a median MELD-score of 7 pts. (IQR 5–10). Most of the patients were classified as ASA class III (70%) and the median dose of propofol infusion was 130 mg (IQR 100–200).

**Table 1:** Demographic and baseline clinical characteristics

Characteristic	
Age—year	56 (49, 63)
Male sex—*n* (%)	23 (62%)
Body weight—kg	71 (65, 85)
Body mass index—kg/m^2^	24.2 (22.4, 28.1)
Child–Pugh—A/B/C	18/18/1
MELD Score—pts	7 (5,10)
Liver enzyme levels—U/L	
Aspartate aminotransferase	70 (48, 109)
Alanine aminotransferase	47 (28, 67)
Etiology of liver cirrhosis—*n* (%)	
ALD	20 (54%)
HCV	8 (22%)
HBV	2 (5%)
Others	7 (19%)
ASA class—(II/III)	11/26

Data are presented as median (IQR) if not otherwise specified

*MELD* model end stage liver disease, *ALD* alcoholic liver disease, *HCV* hepatitis C, *NAFLD* non-alcoholic fatty liver disease

### Hemodynamic changes with propofol sedation

**Fig. 1:**
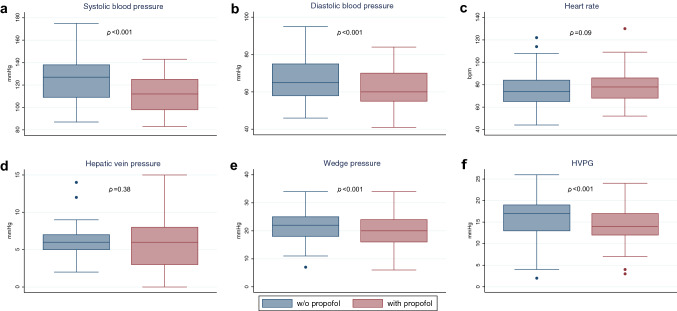
Box plots of hemodynamic parameters with (red) and without (blue) sedation with propofol. Each box signifies the upper and lower quartiles; the median is represented by a line within the box. Whiskers represent the upper and lower adjacent values; outliers are depicted as dots outside the box. Systolic blood pressure values (**a**) and diastolic blood pressure values (**b**) decrease significantly with propofol, while heart rate (**c**) is unchanged. Free hepatic vein pressure (**d**) is not affected by propofol sedation, however, wedged hepatic vein pressure (**e**) and hepatic venous pressure gradient (HVPG) (**f**) significantly decreased with intravenous application of propofol

**Fig. 2:**
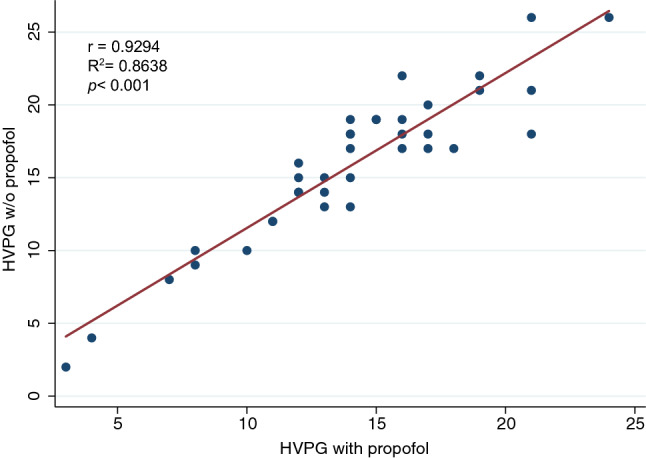
Scatter plot with fitted linear regression line of hepatic venous portal gradient pressure values with and without propofol showing a strong correlation. *HVPG* hepatic venous portal gradient, *w/o* without

**Fig. 3:**
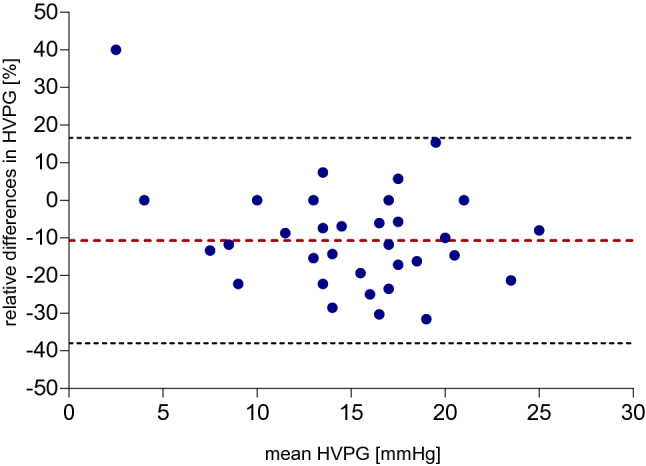
Bland–Altman graph of difference in HVPG measurements with or without propofol sedation. The middle line represents the mean difference of − 10.7% between the two procedures. The two outer dashed lines represent the 95% limits of agreement (lower limit − 38.0, upper limit 16.6)

While patients had normal blood pressure and pulse without sedation, application of propofol led to significant declines in both systolic blood pressure by 12.65 mmHg (95% CI − 17.28 to − 8.02; *p* < 0.001) and diastolic blood pressure by 5.78 mmHg (95% CI − 8.88 to − 2.68; *p* < 0.001) (Fig. 1). Since patients received supplemental oxygen during propofol sedation, oxygen saturation rose by approximately 1% (95% CI 0.28 to 1.66; *p* < 0.007). While measures of free hepatic vein pressure (FHVP) were not altered after propofol sedation (*p* = 0.34), wedged hepatic vein pressure values (WHVP) decreased by 2.05 mmHg (95% CI − 2.46 to − 1.16; *p* < 0.001). There was a strong correlation between HVPG results with and without propofol (*r* = 0.9294, *R*^2^ = 0.8638, *p* < 0.001) (Fig. 2). However, HVPG values obtained under sedation with propofol were in average reduced by 1.8 mmHg (95% CI − 2.46 to − 1.16; *p* < 0.001) when compared to measurements without sedation (Table 2). The difference between the measurements was higher with increasing HVPG values, resulting in a relative difference of − 10.7% (95% CI − 38.01 to 16.63) (Fig. 3) with sedation. There was a weak but significant correlation between the change in HVPG with changes in systemic pressures (change in systolic blood pressure: *r* = 0.3417, *R*^2^ = 0.1167, *p* < 0.04; change in diastolic blood pressure: *r* = 0.5222, *R*^2^ = 0.2727, *p* < 0.001; change in mean arterial pressure: *r* = 0.4950, *R*^2^ = 0.2450, *p* < 0.002). There was no difference in the effect of propofol when patients were stratified according to CHILD category or etiology of cirrhosis (Supplementary Appendix Fig. S1, Fig. S2 and Fig. S3). There was no dose relationship of propofol with the relative change in HVPG (*r* = 0.1081, *R*^2^ = 0.0117, *p* = 0.52; Supplementary Appendix Fig. S4).

**Table 2:** Hemodynamic parameters w/o and with propofol sedation

	w/o Propofol	With propofol	Difference (95% CI)	*p* Value
Systolic blood pressure [mmHg]	125 (19.6; 87–175)	112 (17.1; 83–143)	− 12.65 (95% CI − 17.28 to − 8.02)	< 0.001
Diastolic blood pressure [mmHg]	67 (12.3; 46–95)	61 (11.0; 41–84)	− 5.78 (95% CI − 8.88 to − 2.68)	< 0.001
Heart rate [bpm]	77 (17.6; 44–122)	79 (15.9; 52–130)	1.86 (95% CI − 0.27 to 4.00)	0.09
Oxygen saturation [%]	98 (2.2; 93–100)	99 (1.2; 95–100)	0.97 (95% CI, 0.28–1.66)	0.007
Free hepatic vein pressure (FHVP) [mmHg]	6.1 (2.5; 2–14)	5.8 (3.1; 0–15)	− 0.24 (95% CI − 0.81 to 0.32)	0.39
Wedged hepatic vein pressure (WHVP) [mmHg]	21.9 (5.9; 7–34)	19.9 (5.8; 6–34)	− 2.05 (95% CI − 2.74 to − 1.37)	< 0.001
Hepatic vein pressure gradient (HVPG) [mmHg]	15.9 (5.2; 2–26)	14.1 (4.6; 3–24)	− 1.81 (95% CI − 2.46 to − 1.16)	< 0.001

Absolute values are presented as mean (SD; range). Hepatic vein pressure, wedge pressure and HVPG are mean estimates derived from three subsequent measurements

*FHVP* free hepatic vein pressure, *HVGP* hepatic vein pressure gradient, *WHVP* wedged hepatic vein pressure, *w/o* without

### Diagnostic accuracy of HVPG measurement under propofol sedation

HVPG measurement under propofol sedation showed an overall very good intra-class correlation coefficient of 0.927 (95% CI 0.594–0.975).

In total, 33 (89.2%) of 37 patients had CSPH with a HVPG of 10 mmHg or higher. Under sedation with propofol, one patient (2.7%) was incorrectly classified due to a decrease of HVPG from 10 mmHg without propofol to 8 mmHg with propofol (Table 3). Measurement of HVPG under propofol using the clinical decision cutoff at 10 mmHg was associated with an area under the curve of 0.985 (95% CI 0.96 to 1.00), a sensitivity of 0.969 and a specificity of 1.0.

**Table 3:** Proportions of patients with clinically significant portal hypertension (HVPG ≥ 10 mmHg)

w/o Propofol	With propofol		Total
	HVPG < 10 mmHg	HVPG ≥ 10 mmHg	
HVPG < 10 mmHg	4	0	4
HVPG ≥ 10 mmHg	1	32	33
Total	5	32	37

4 × 4 table with proportions of patients categorized as “no significant portal hypertension” with HVPG < 10 mmHg or “CSPH” with HVPG ≥ 10 mmHg

*CSPH* clinically significant portal hypertension, *HVGP* hepatic vein pressure gradient, *w/o* without

Portal hypertension with HVPG of 12 mmHg or higher was found in 31 (83.8%) out of 37 patients. Under sedation with propofol, two patients (5.4%) were incorrectly classified due to a decrease of HVPG in both patients from 12 mmHg without propofol to 11 mmHg with propofol (Table 4). Measurement of HVPG under propofol using the clinical decision cutoff at 12 mmHg was associated with an area under the curve of 0.935 (95% CI 0.92 to 1.00), a sensitivity of 0.935 and a specificity of 1.0.

**Table 4:** Proportions of patients with significant portal hypertension (HVPG ≥ 12 mmHg)

w/o Propofol	With propofol		Total
	HVPG < 12 mmHg	HVPG ≥ 12 mmHg	
HVPG < 12 mmHg	6	0	6
HVPG ≥ 12 mmHg	2	29	31
Total	8	29	37

4 × 4 table with proportions of patients categorized as “HVPG < 12 mmHg” or “HVPG ≥ 10 mmHg”

*HVGP* hepatic vein pressure gradient, *w/o* without

When HVPG values were adjusted for the systematic error of around 10% lower results via division by 0.9, all patients were correctly classified at a cutoff of 12 mmHg.

## Discussion

In this prospective observational study of patients with cirrhosis and suspected portal hypertension undergoing HVPG measurement, sedation with propofol considerably affected HVPG readings which might have significant implications on clinical decision-making especially in cases with borderline values.

Propofol is the standard intravenous sedative drug for short procedures in digestive and liver diseases which is mainly explained by its short time to onset of action as well as short recovery time [12, 17, 18]. Propofol can safely be administered by non-anesthesiologists with very low rates of sedation-associated adverse events. However, propofol is known to cause relevant hemodynamic depressant effects with decreases in cardiac index and stroke volume index as well as peripheral vasodilatation, which all may contribute to hypotension [19–21]. It is, therefore, to be assumed that it may have relevant effects on portal pressure values as well. For this reason, measurements of HVPG have consistently been performed without sedation.

In our study, propofol sedation led to a reduction of wedged hepatic vein pressures without affecting the free hepatic vein pressure values, mirroring an effect of propofol on a decreased splanchnic blood flow. The difference between awake condition and during sedation was around − 10%; however, there was a rather broad variation which was independent from the dose of propofol. Overall, HVPG measurement under propofol sedation had a very good intra-class correlation coefficient of 0.927. In total, 5.4% of patients—all patients with borderline values—would have been misclassified with propofol sedation if not adjusted for the average reduction by 10%. Though, after correction all patients would have been correctly classified as < 12 mmHg vs. ≥ 12 mmHg, which is an accepted cutoff to guide clinical decision-making [8, 16, 22].

In a previous study by Reverter et al. [23] the impact of deep sedation on HVPG measurements was investigated with a combination of propofol with remifentanil. The combination of these drugs led to a marked decrease in respiratory rate, which was associated with much deeper and strained breaths resulting in relevant oscillations in both cardio-pulmonary, and hepatic venous pressures. Both FHVP and WHVP measures oscillated by around 5 mmHg with a range of up to 36.5 mmHg due to progressive increase and decrease of intra-abdominal pressure during prolonged and strained breaths. As a consequence of these large oscillations, around half of the patients with the deep sedation would have been misclassified in acute response to i.v. propranolol [23]. In opposite to these results, in our study with only moderate sedation using propofol only, we did not observe comparable oscillations, neither in cardiovascular nor in HVPG values. However, it remains unclear whether respiratory oscillations in the previous study were primarily driven by the additional administration of remifentanil or due to the aimed level of sedation.

All HVPG measures in our study were determined as triplicates and the single values did not vary from the mean to a higher extent than observed in awake conditions (≤ 1 mmHg). Nevertheless, respiratory oscillations might be of high relevance in patients with known obstructive sleep apnea who have a higher risk of hypoxemia and strained respiration during propofol sedation [18]. Therefore, patients with difficult airways may not qualify for HVPG measurement with sedation.

In recent years, several non-invasive techniques have been proposed for the determination of portal hypertension: liver stiffness measured by transient elastography [24, 25], collaterals on imaging [5], magnetic resonance elastography [26], or combinations of several non-invasive tests have been assessed as possible alternatives to invasive HVPG measurement; however, thus far, none has proved sufficiently accurate to replace the current gold-standard method of HVPG measurement. While further research is needed to find a non-invasive alternative to the current standard practice, sedation might be an easy and convenient tool to offer HVPG measurement to a wider population of patients, especially those who are anxious or unwilling to undergo the procedure. However, even propofol monotherapy leads to changes in HVPG, therefore, sedation should be reserved for special cases with a strong demand for sedation despite patient education, possibly taking into the account the correction factor. Another factor to be taken into account is the investigation of the response to non-selective beta blocker (NSBB) treatment as assessed in paired HVPG measurements. The cut-offs defining HVPG-response to NSBB treatment (primary prophylaxis) or response to etiological therapy has been accepted at − 10%. Looking at the variations that may be introduced by propofol sedation, different doses between investigations, and variations in sedation depth, response testing to NSBB may be significantly altered by propofol sedation. As a consequence, propofol sedation does not seem to be an option, if hemodynamic response assessment (NSBB or etiological therapies) is intended.

In an older study, HVPG measurements have been investigated with the use of different doses of the short-acting benzodiazepine midazolam [11]. At a dose of 0.02 mg/kg, it did not alter HVPG values, but achieved significantly increased patient comfort and relaxation during the procedure. Hence, midazolam may be an alternative sedative agent in patients with anxiety and unwillingness to undergo HVPG measurement, but higher doses should not be used. Nevertheless, the measurement of HVPG is a minimally invasive procedure and generally well tolerated, thus to obtain unaffected measures patients should be motivated to undergo the procedure without sedative agents.

## Supplementary Information

Below is the link to the electronic supplementary material.

Supplementary file1 (DOCX 25 KB)

## Data Availability

The data that support the findings of this study are available from the corresponding author, upon reasonable request.
