# Supplementary material for: Impact of propofol sedation on the diagnostic accuracy of hepatic venous pressure gradient measurements in patients with cirrhosis
Source: Hepatol Int. 2021 Oct 26;16(4):817–23. doi: 10.1007/s12072-021-10261-z (PMC9349095; doi:10.1007/s12072-021-10261-z)
Supplement: Supplementary file 1 — Supplementary file1 (DOCX 25 KB) [file 12072_2021_10261_MOESM1_ESM.docx]

Supplementary Appendix

This appendix has been provided by the authors to give readers additional information about their work.

Supplement to: Ebrahimi F, Semela D and Heim M. *Impact of Propofol Sedation on the Diagnostic Accuracy of Hepatic Venous Pressure Gradient Measurements in Patients with Cirrhosis*.

**Figure S1 – Effect of propofol on hepatic venous pressure gradient (HVPG) stratified by CHILD category**

**Figure S2 – Effect of propofol on wedged hepatic vein pressure (WHVP) stratified by CHILD category**

**Figure S3 – Effect of propofol on hepatic venous pressure gradient (HVPG) stratified by etiology**

ALD, alcoholic liver disease; HCV, hepatitis C virus; HBV, hepatitis B virus

**Figure S4 – Scatter Plot of Scatter Plot of Change in HVPG and Propofol dose (in mg)**
